# Probabilistic Hierarchical Quantum Information Splitting of Arbitrary Multi-Qubit States

**DOI:** 10.3390/e24081077

**Published:** 2022-08-04

**Authors:** Jie Tang, Song-Ya Ma, Qi Li

**Affiliations:** 1 School of Mathematics and Statistics, Henan University, Kaifeng 475004, China; 2 Information Security Center, State Key Laboratory of Networking and Switching Technology, Beijing University of Posts and Telecommunications, Beijing 100876, China; 3 Guangxi Key Laboratory of Trusted Software, Guilin University of Electronic Technology, Guilin 541004, China

**Keywords:** hierarchical quantum information splitting, non-maximally entangled cluster state, multi-qubit state, POVM, recovery operation

## Abstract

By utilizing the non-maximally entangled four-qubit cluster states as the quantum channel, we first propose a hierarchical quantum information splitting scheme of arbitrary three-qubit states among three agents with a certain probability. Then we generalize the scheme to arbitrary multi-qubit states. Hierarchy is reflected on the different abilities of agents to restore the target state. The high-grade agent only needs the help of one low-grade agent, while the low-grade agent requires all the other agents’ assistance. The designated receiver performs positive operator-valued measurement (POVM) which is elaborately constructed with the aid of Hadamard matrix. It is worth mentioning that a general expression of recovery operation is derived to disclose the relationship with measurement outcomes. Moreover, the scheme is extended to multiple agents by means of the symmetry of cluster states.

## 1. Introduction

With the rapid development of computer networks, the demand for information security is sharply increasing. Quantum cryptography has unconditional security, which depends on the internal physical characteristics of quantum mechanics, such as the Heisenberg uncertainty principle and non-cloning theorem [1]. As the counterpart of classical secret sharing [2], quantum secret sharing (QSS) was first introduced by Hillery et al. [3] and has been one of the most significant branches of quantum cryptography. In QSS, the secret is divided into multiple pieces (called shares) and it can be constructed only through the cooperation of the shares. When the shared secret is a quantum state, QSS is termed quantum state sharing (QSTS) or quantum information splitting (QIS). The other agents can be regarded as the controlling party if one agent is identified as the receiver. From this point of view, QIS can be used to complete the task of controlled quantum teleportation (CQT) [4] in which an unknown state is teleported from a sender to a spatially separated receiver under the supervision of one or more controllers. Owing to its potential applications, e.g., creating joint accounts containing quantum money [5] and secure distributed quantum computation [6], QIS has been intensively studied [7,8,9,10,11,12,13,14,15] in the past two decades. For example, Luo et al. [11] investigated the application of χ state for QIS of an arbitrary three-qubit state. Li et al. [12] proposed a novel class of universal and flexible QIS schemes of arbitrary qubit and qudit states using quantum walks with multiple coins. In the meantime, experimental implementation of QIS has been reported [16,17,18].

The QIS schemes mentioned above mainly focus on the situation in which the authorities of the agents are identical. Considering the actual communication circumstance, Wang et al. [19] first proposed a hierarchical quantum information splitting (HQIS) scheme in the case where the agents are graded according to their abilities to restore the secret state. The high-grade agent requires some of the other agents’ help to complete the task, while the low-grade agent can do it only if all the other agents supply the assistance. Since then, much attention [20,21,22,23,24,25] has been paid to HQIS due to its useful applications in practice. Like many other quantum communication schemes [26,27,28], HQIS prefers using maximal entanglement to achieve perfect transmission. Nevertheless, the maximally entangled states are hard to maintain owing to the decoherence induced by the surrounding environment [29]. Thus, it is of great importance to investigate quantum communication via non-maximally entangled (NME) states [30,31,32,33,34,35,36]. Recently, based on NME four-particle cluster states, Xu et al. [35] and Guo et al. [36] respectively sketched new HQIS protocols to probabilistically realize the QSS of arbitrary unknown single-qubit state and two-qubit state via POVM. To our knowledge, there are no universal HQIS schemes of arbitrary multi-qubit states via POVM.

In this paper, we intend to devise a universal scheme to achieve the HQIS of arbitrary multi-qubit states with three agents via the NME resource. The agents lie in two disparate grades of which one agent lies in the high grade and two agents lie in the low grade. With the aid of the Hadamard matrix, we construct the general POVM operators. The recovery operation is derived from a general expression which distinctly discloses the relationship with measurement results. Based on the assistance of one inferior agent, the high-grade agent as the designated receiver can recover the target state with a certain probability by performing POVM rather than the usual projective measurement. While the low-grade agent requires all agents’ help. Then, we generalize the scheme from three agents to multiple agents by means of the symmetry of cluster states.

The outline of this paper is organized as follows. In Section 2, we first put forward a probabilistic HQIS scheme of arbitrary three-qubit states with three agents by utilizing the NME four-qubit cluster states as the entangled resource. The above scheme is extended to arbitrary *n*-qubit states in Section 3. In Section 4, we generalize the universal scheme of multi-qubit states from three agents to multiple agents. Section 5 is the security analysis. Some discussions and conclusions are given in the last section.

## 2. Probabilistic HQIS of Arbitrary Three-Qubit States with Three Agents

There are four participants, Alice, Bob, Charlie_1_ and Charlie_2_. Alice is the sender who wishes to teleport an unknown state to three agents in an asymmetric way such that any one of them can restore the secret state under the cooperation of other agents. The three agents are divided into two grades according to their abilities to recover the target state. High-grade agent Bob can restore the secret state only with the help of one low-grade agent while low-grade agent Charlie_1_ or Charlie_2_ needs the assistance of all the other agents.

Alice possesses a secret three-qubit state:

(1) $$\begin{array}{cc} {|\varphi \rangle }_{123}= & (\alpha_{0}|000\rangle +\alpha_{1}|001\rangle +\alpha_{2}|010\rangle +\alpha_{3}|011\rangle \\ & +\alpha_{4}|100\rangle +\alpha_{5}|101\rangle +\alpha_{6}|110\rangle +\alpha_{7}{\left|111\rangle \right)}_{123}. \end{array}$$

She knows nothing about the state except that the complex parameters $\alpha_{0},\cdots ,\alpha_{7}$ satisfy the normalization condition $\sum_{j=0}^{7}{\left|\alpha_{j}\right|}^{2}=1$.

Three NME four-qubit cluster states,

(2) $$|\Psi_{j}\rangle_{A_{j}B_{j}C_{1j}C_{2j}}=(\beta_{j0}|0\rangle |\psi^{0}\rangle +\beta_{j1}\left|1\rangle \right|\psi^{1}{\rangle )}_{A_{j}B_{j}C_{1j}C_{2j}},\;j=1,2,3$$

are selected as entangled resource, where

(3) $$|\psi^{0}\rangle =|000\rangle +|011\rangle ,\;|\psi^{1}\rangle =|100\rangle -|111\rangle ,$$

$\beta_{j0},\beta_{j1}$ are nonzero real numbers satisfying $\beta_{j0}^{2}+\beta_{j1}^{2}=\frac{1}{2}$ and $|\beta_{j0}\left|<\right|\beta_{j1}|$. The qubit $A_{j}$ is held by Alice, qubits $B_{j}$, $C_{1j}$ and $C_{2j}$ are distributed to the agents Bob, Charlie_1_ and Charlie_2_, respectively.

The initial whole system can be expressed as:

(4) $$\begin{array}{cc} & {|\varphi \rangle }_{123}⊗|\Psi_{1}\rangle_{A_{1}B_{1}C_{11}C_{21}}⊗|\Psi_{2}\rangle_{A_{2}B_{2}C_{12}C_{22}}⊗{|\Psi_{3}\rangle }_{A_{3}B_{3}C_{13}C_{23}}\\ = & (\alpha_{0}|000\rangle +\alpha_{1}|001\rangle +\alpha_{2}|010\rangle +\alpha_{3}|011\rangle +\alpha_{4}|100\rangle +\alpha_{5}|101\rangle +\alpha_{6}|110\rangle \\ & +\alpha_{7}{\left|111\rangle \right)}_{123}(\sum_{p_{1},p_{2},p_{3}=0}^{1}\beta_{p}|p_{1}p_{2}p_{3}\rangle |\psi^{p_{1}}\psi^{p_{2}}\psi^{p_{3}}{\rangle )}_{A_{1}A_{2}A_{3}B_{1}C_{11}C_{21}B_{2}C_{12}C_{22}B_{3}C_{13}C_{23}}. \end{array}$$

For convenience, we denote $\beta_{1p_{1}}\beta_{2p_{2}}\beta_{3p_{3}}=\beta_{p}$, where *p* is the decimal form of the binary string $p_{1}p_{2}p_{3}$. In other words, $p=p_{1}2^{2}+p_{2}2^{1}+p_{3}2^{0}$.

To complete the task, the participants need to perform the following operations.

**Step 1** The sender Alice performs two-qubit projective measurements on her qubits $\left(1,A_{1}\right)$, $\left(2,A_{2}\right)$, $\left(3,A_{3}\right)$ under Bell basis,

(5) $$|\xi_{00}\rangle =\frac{|00\rangle +|11\rangle }{\sqrt{2}},|\xi_{01}\rangle =\frac{|01\rangle +|10\rangle }{\sqrt{2}},|\xi_{10}\rangle =\frac{|00\rangle -|11\rangle }{\sqrt{2}},|\xi_{11}\rangle =\frac{|01\rangle -|10\rangle }{\sqrt{2}}.$$

After the measurements, she broadcasts 6 bit classical information $s_{1}t_{1}s_{2}t_{2}s_{3}t_{3}$ corresponding to her measurement outcome ${|\xi_{s_{1}t_{1}}\rangle }_{1A_{1}}{|\xi_{s_{2}t_{2}}\rangle }_{2A_{2}}{|\xi_{s_{3}t_{3}}\rangle }_{3A_{3}}$, $s_{j},t_{j}=0,1,j=1,2,3$.

As a result, the remaining state collapses with equal probability into one of the 64 states $|g_{s_{1}t_{1}s_{2}t_{2}s_{3}t_{3}}\rangle$:

(6) $$\begin{array}{c} \frac{1}{2\sqrt{2}}\{\sum_{j=0}^{1}{\left(-1\right)}^{js_{1}}\beta_{1,t_{1}⊕j}|\psi^{t_{1}⊕j}\rangle [\alpha_{4j}\beta_{2,t_{2}}\beta_{3,t_{3}}|\psi^{t_{2}}\psi^{t_{3}}\rangle +{\left(-1\right)}^{s_{3}}\alpha_{4j+1}\beta_{2,t_{2}}\beta_{3,t_{3}⊕1}|\psi^{t_{2}}\psi^{t_{3}⊕1}\rangle \\ +{\left(-1\right)}^{s_{2}}\alpha_{4j+2}\beta_{2,t_{2}⊕1}\beta_{3,t_{3}}|\psi^{t_{2}⊕1}\psi^{t_{3}}{\rangle +\left(-1\right)}^{s_{2}⊕s_{3}}\alpha_{4j+3}\beta_{2,t_{2}⊕1}\beta_{3,t_{3}⊕1}|\psi^{t_{2}⊕1}\psi^{t_{3}⊕1}\rangle ]\}. \end{array}$$

Here and hereafter ⊕ means modulo 2 addition.

In order to clearly explain how our protocol implements, suppose Alice’s measurement result is $|\xi_{11}\rangle_{1A_{1}}$$|\xi_{01}\rangle_{2A_{2}}$$|\xi_{10}\rangle_{3A_{3}}$. The three agents’ qubits $\left(B_{1},C_{11},C_{21},B_{2},C_{12},C_{22},B_{3},C_{13},C_{23}\right)$ become

(7) $$\begin{array}{cc} |g_{110110}\rangle = & \frac{1}{2\sqrt{2}}(\alpha_{0}\beta_{3}|\psi^{0}\psi^{1}\psi^{1}\rangle +\alpha_{1}\beta_{2}|\psi^{0}\psi^{1}\psi^{0}\rangle -\alpha_{2}\beta_{1}|\psi^{0}\psi^{0}\psi^{1}\rangle -\alpha_{3}\beta_{0}|\psi^{0}\psi^{0}\psi^{0}\rangle \\ & +\alpha_{4}\beta_{7}|\psi^{1}\psi^{1}\psi^{1}\rangle +\alpha_{5}\beta_{6}|\psi^{1}\psi^{1}\psi^{0}\rangle -\alpha_{6}\beta_{5}|\psi^{1}\psi^{0}\psi^{1}\rangle -\alpha_{7}\beta_{4}|\psi^{1}\psi^{0}\psi^{0}\rangle ). \end{array}$$

At this time, none of the agents can recover the target state privately since their qubits are entangled with each other. Other agents’ cooperation is indispensable for the designated agent to complete his recovery task.

**Step 2** According to Alice’s classical message, the agents apply the appropriate operations to achieve the goal that any one of agents recovers the secret state.

It will be discussed in two cases as far as the agents’ different authorities are concerned.

**Case 1** The high-grade agent is designated as the receiver.

The state in Equation (7) can be decomposed as:

(8) $$\begin{array}{ccc} |g_{110110}\rangle & = & \frac{1}{2\sqrt{2}}{[|000\rangle }^{⊗2}f\left(-\alpha_{6},\alpha_{7},-\alpha_{4},\alpha_{5},\alpha_{2},-\alpha_{3},\alpha_{0},-\alpha_{1}\right)\\ & & +{|001\rangle }^{⊗2}f\left(-\alpha_{6},-\alpha_{7},-\alpha_{4},-\alpha_{5},\alpha_{2},\alpha_{3},\alpha_{0},\alpha_{1}\right)\\ & & +{|010\rangle }^{⊗2}f\left(-\alpha_{6},\alpha_{7},\alpha_{4},-\alpha_{5},\alpha_{2},-\alpha_{3},-\alpha_{0},\alpha_{1}\right)\\ & & +{|011\rangle }^{⊗2}f\left(-\alpha_{6},-\alpha_{7},\alpha_{4},\alpha_{5},\alpha_{2},\alpha_{3},-\alpha_{0},-\alpha_{1}\right)\\ & & +{|100\rangle }^{⊗2}f\left(-\alpha_{6},\alpha_{7},-\alpha_{4},\alpha_{5},-\alpha_{2},\alpha_{3},-\alpha_{0},\alpha_{1}\right)\\ & & +{|101\rangle }^{⊗2}f\left(-\alpha_{6},-\alpha_{7},-\alpha_{4},-\alpha_{5},-\alpha_{2},-\alpha_{3},-\alpha_{0},-\alpha_{1}\right)\\ & & +{|110\rangle }^{⊗2}f\left(-\alpha_{6},\alpha_{7},\alpha_{4},-\alpha_{5},-\alpha_{2},\alpha_{3},\alpha_{0},-\alpha_{1}\right)\\ & & +{|111\rangle }^{⊗2}f\left(-\alpha_{6},-\alpha_{7},\alpha_{4},\alpha_{5},-\alpha_{2},-\alpha_{3},\alpha_{0},\alpha_{1}\right){]}_{C_{11}C_{12}C_{13}C_{21}C_{22}C_{23}B_{1}B_{2}B_{3}}, \end{array}$$

where

(9) $$\begin{array}{cc} f(u_{0},\cdots ,u_{7})= & u_{0}\beta_{0}|000\rangle +u_{1}\beta_{1}|001\rangle +u_{2}\beta_{2}|010\rangle +u_{3}\beta_{3}|011\rangle \\ & +u_{4}\beta_{4}|100\rangle +u_{5}\beta_{5}|101\rangle +u_{6}\beta_{6}|110\rangle +u_{7}\beta_{7}|111\rangle . \end{array}$$

It is clear that Charlie_1_’s and Charlie_2_’s qubits are identical. From Equation (8), if one of them carries out single-qubit projective measurements under *Z*-basis {|0〉,|1〉}, the qubits of Bob and the other low-grade agent will collapse into a product state. That means only one of the low-grade agents is sufficient to supply assistance. Suppose Charlie_1_ performs projective measurements and gets result ${|0\rangle }_{C_{11}}{|1\rangle }_{C_{12}}{|0\rangle }_{C_{13}}$, which is sent to Bob in the form of classical message 010. Then Bob’s qubits collapse into:

(10) $$\begin{array}{c} \frac{1}{2\sqrt{2}}(-\alpha_{0}\beta_{6}|110\rangle +\alpha_{1}\beta_{7}|111\rangle +\alpha_{2}\beta_{4}|100\rangle -\alpha_{3}\beta_{5}|101\rangle \\ +\alpha_{4}\beta_{2}|010\rangle -\alpha_{5}\beta_{3}|011\rangle -\alpha_{6}\beta_{0}|000\rangle +\alpha_{7}\beta_{1}{\left|001\rangle \right)}_{B_{1}B_{2}B_{3}}. \end{array}$$

One can see that Bob’s state mix the information of the secret state and the entangled channel. To reconstruct the secret state, Bob introduces three auxiliary qubits with initial states ${|000\rangle }_{e_{1}e_{2}e_{3}}$ and executes Controlled-NOT (CNOT) operations $C_{B_{j}e_{j}}$ with the qubit $B_{j}$ as the controlled qubit and $e_{j}$ as the target one, j=1,2,3. Thus, Bob’s state transforms into:

(11) $$\begin{array}{cc} |\Gamma \rangle = & \frac{1}{2\sqrt{2}}{(-\alpha_{0}\beta_{6}|110\rangle }^{⊗2}+\alpha_{1}\beta_{7}{|111\rangle }^{⊗2}+\alpha_{2}\beta_{4}{|100\rangle }^{⊗2}-\alpha_{3}\beta_{5}{|101\rangle }^{⊗2}\\ & +\alpha_{4}\beta_{2}{|010\rangle }^{⊗2}-\alpha_{5}\beta_{3}{|011\rangle }^{⊗2}-\alpha_{6}\beta_{0}{|000\rangle }^{⊗2}+\alpha_{7}\beta_{1}{|001\rangle }^{⊗2}{)}_{B_{1}B_{2}B_{3}e_{1}e_{2}e_{3}}. \end{array}$$

Denote

(12) $$\begin{array}{c} \left(\begin{array}{c} |K_{000}\rangle \\ |K_{001}\rangle \\ |K_{010}\rangle \\ |K_{011}\rangle \\ |K_{100}\rangle \\ |K_{101}\rangle \\ |K_{110}\rangle \\ |K_{111}\rangle \end{array}\right)=H^{⊗3}\cdot \mathrm{diag}\left\{-\alpha_{6},\alpha_{7},\alpha_{4},-\alpha_{5},\alpha_{2},-\alpha_{3},-\alpha_{0},\alpha_{1}\right\}\left(\begin{array}{c} |000\rangle \\ |001\rangle \\ |010\rangle \\ |011\rangle \\ |100\rangle \\ |101\rangle \\ |110\rangle \\ |111\rangle \end{array}\right) \end{array}$$

and

(13) $$\begin{array}{c} \left(\begin{array}{c} |Q_{000}\rangle \\ |Q_{001}\rangle \\ |Q_{010}\rangle \\ |Q_{011}\rangle \\ |Q_{100}\rangle \\ |Q_{101}\rangle \\ |Q_{110}\rangle \\ |Q_{111}\rangle \end{array}\right)=H^{⊗3}\cdot \mathrm{diag}\left\{\beta_{0},\beta_{1},\beta_{2},\beta_{3},\beta_{4},\beta_{5},\beta_{6},\beta_{7}\right\}\left(\begin{array}{c} |000\rangle \\ |001\rangle \\ |010\rangle \\ |011\rangle \\ |100\rangle \\ |101\rangle \\ |110\rangle \\ |111\rangle \end{array}\right), \end{array}$$

where *H* is the Hadamard matrix $\left(\begin{array}{cc} 1 & 1\\ 1 & -1 \end{array}\right)$. Then Equation (11) can be rephrased as:

(14) $$|\Gamma \rangle =\frac{1}{16\sqrt{2}}\sum_{r_{1},r_{2},r_{3}=0}^{1}{|K_{r_{1}r_{2}r_{3}}\rangle }_{B_{1}B_{2}B_{3}}{|Q_{r_{1}r_{2}r_{3}}\rangle }_{e_{1}e_{2}e_{3}}.$$

It shows that $|K_{r_{1}r_{2}r_{3}}\rangle$ can be obtained if performing appropriate measurements on the auxiliary qubits and obtains the measurement result $|Q_{r_{1}r_{2}r_{3}}\rangle$. Unfortunately, $|Q_{000}\rangle ,\cdots ,|Q_{111}\rangle$ are not mutually orthogonal such that they cannot be distinguished deterministically by the usual projective measurement. To differentiate non-orthogonal states, Bob needs to execute POVM on his auxiliary qubits under measurement operators $\left\{O_{000},\cdots ,O_{111},O_{8}\right\}$:

(15) $$O_{r_{1}r_{2}r_{3}}=\frac{1}{\omega }|M_{r_{1}r_{2}r_{3}}\rangle \langle M_{r_{1}r_{2}r_{3}}|,\;O_{8}=I-\sum_{r_{1},r_{2},r_{3}=0}^{1}O_{r_{1}r_{2}r_{3}},$$

where ω is a coefficient related with $\beta_{0},\cdots ,\beta_{7}$ and

(16) $$\begin{array}{c} \left(\begin{array}{c} |M_{000}\rangle \\ |M_{001}\rangle \\ |M_{010}\rangle \\ |M_{011}\rangle \\ |M_{100}\rangle \\ |M_{101}\rangle \\ |M_{110}\rangle \\ |M_{111}\rangle \end{array}\right)=\frac{1}{\sqrt{\varepsilon }}H^{⊗3}\cdot \mathrm{diag}\left\{\frac{1}{\beta_{0}},\frac{1}{\beta_{1}},\frac{1}{\beta_{2}},\frac{1}{\beta_{3}},\frac{1}{\beta_{4}},\frac{1}{\beta_{5}},\frac{1}{\beta_{6}},\frac{1}{\beta_{7}}\right\}\left(\begin{array}{c} |000\rangle \\ |001\rangle \\ |010\rangle \\ |011\rangle \\ |100\rangle \\ |101\rangle \\ |110\rangle \\ |111\rangle \end{array}\right). \end{array}$$

Here, $\varepsilon =\sum_{j=0}^{7}\frac{1}{\beta_{j}^{2}}=\frac{1}{8\beta_{10}^{2}\beta_{11}^{2}\beta_{20}^{2}\beta_{21}^{2}\beta_{30}^{2}\beta_{31}^{2}}$. In order to ensure operator $O_{8}$ is positive, ω should satisfy $\omega \geq 64\max\{\beta_{0}^{2},\cdots ,\beta_{7}^{2}\}$.

If Bob obtains the measurement result $O_{r_{1}r_{2}r_{3}}$ with the probability $\langle \Gamma |O_{r_{1}r_{2}r_{3}}|\Gamma \rangle =\frac{1}{8\omega \varepsilon }$, he can restore the target state by performing appropriate Pauli operations. For clarity, assume his measurement result is $O_{011}$. Then Bob performs operation $Y_{{}_{B_{1}}}X_{{}_{B_{2}}}$$I_{{}_{B_{3}}}$ on his collapsed state

(17) $$\begin{array}{cc} |K_{011}\rangle = & (\alpha_{0}|110\rangle +\alpha_{1}|111\rangle +\alpha_{2}|100\rangle +\alpha_{3}|101\rangle \\ & -\alpha_{4}|010\rangle -\alpha_{5}|011\rangle -\alpha_{6}|000\rangle -\alpha_{7}{\left|001\rangle \right)}_{B_{1}B_{2}B_{3}} \end{array}$$

and recovers the target state. Bob’s recovery operations conditioned on Charlie_1_’s (or Charlie_2_’s) measurement result and his own POVM outcome $O_{r_{1}r_{2}r_{3}}$ are summarized in Table 1, where *I*, *X*, *Y*, *Z* are the Pauli operations. However, Bob may also get $O_{8}$ with the probability $1-\frac{1}{\omega \varepsilon }$. In this case, he cannot infer the secret state of his qubits. Therefore, the total success probability is:

(18) $$P=\frac{1}{8\omega \varepsilon }\times 8\times 8\times 64=\frac{{\left(64\beta_{10}\beta_{11}\beta_{20}\beta_{21}\beta_{30}\beta_{31}\right)}^{2}}{\omega }.$$

**Case 2** The low-grade agent is designated as the receiver.

Suppose Charlie_1_ is appointed as the receiver since Charlie_1_ and Charlie_2_ have the same authority.

Denote

(19) $$|\tilde{0}\rangle =|-\rangle =\frac{|0\rangle +|1\rangle }{\sqrt{2}},\;|\tilde{1}\rangle =|-\rangle =\frac{|0\rangle -|1\rangle }{\sqrt{2}}$$

and

(20) $$\begin{array}{ccc} \tilde{f}\left(u_{0},\cdots ,u_{7}\right) & = & u_{0}|+++\rangle +u_{1}|++-\rangle +u_{2}|+-+\rangle +u_{3}|+--\rangle \\ & & +u_{4}|-++\rangle +u_{5}|-+-\rangle +u_{6}|--+\rangle +u_{7}|---\rangle . \end{array}$$

Then the state in Equation (7) can be rewritten as:

(21) $$\begin{array}{cc} |g_{110110}\rangle = & \frac{1}{8}\sum_{b_{1},b_{2},b_{3}=0}^{1}{|\tilde{b_{1}}\tilde{b_{2}}\tilde{b_{3}}\rangle }_{B_{1}B_{2}B_{3}}[|+++\rangle \tilde{f}\left({\hat{\alpha }}_{6},{\hat{\alpha }}_{7},{\hat{\alpha }}_{4},{\hat{\alpha }}_{5},{\hat{\alpha }}_{2},{\hat{\alpha }}_{3},{\hat{\alpha }}_{0},{\hat{\alpha }}_{1}\right)\\ & +|++-\rangle \tilde{f}\left({\hat{\alpha }}_{7},{\hat{\alpha }}_{6},{\hat{\alpha }}_{5},{\hat{\alpha }}_{4},{\hat{\alpha }}_{3},{\hat{\alpha }}_{2},{\hat{\alpha }}_{1},{\hat{\alpha }}_{0}\right)\\ & -|+-+\rangle \tilde{f}\left({\hat{\alpha }}_{4},{\hat{\alpha }}_{5},{\hat{\alpha }}_{6},{\hat{\alpha }}_{7},{\hat{\alpha }}_{0},{\hat{\alpha }}_{1},{\hat{\alpha }}_{2},{\hat{\alpha }}_{3}\right)\\ & -|+--\rangle \tilde{f}\left({\hat{\alpha }}_{5},{\hat{\alpha }}_{4},{\hat{\alpha }}_{7},{\hat{\alpha }}_{6},{\hat{\alpha }}_{1},{\hat{\alpha }}_{0},{\hat{\alpha }}_{3},{\hat{\alpha }}_{2}\right)\\ & -|-++\rangle \tilde{f}\left({\hat{\alpha }}_{2},{\hat{\alpha }}_{3},{\hat{\alpha }}_{0},{\hat{\alpha }}_{1},{\hat{\alpha }}_{6},{\hat{\alpha }}_{7},{\hat{\alpha }}_{4},{\hat{\alpha }}_{5}\right)\\ & -|-+-\rangle \tilde{f}\left({\hat{\alpha }}_{3},{\hat{\alpha }}_{2},{\hat{\alpha }}_{1},{\hat{\alpha }}_{0},{\hat{\alpha }}_{7},{\hat{\alpha }}_{6},{\hat{\alpha }}_{5},{\hat{\alpha }}_{4}\right)\\ & +|--+\rangle \tilde{f}\left({\hat{\alpha }}_{0},{\hat{\alpha }}_{1},{\hat{\alpha }}_{2},{\hat{\alpha }}_{3},{\hat{\alpha }}_{4},{\hat{\alpha }}_{5},{\hat{\alpha }}_{6},{\hat{\alpha }}_{7}\right)\\ & +|---\rangle \tilde{f}\left({\hat{\alpha }}_{1},{\hat{\alpha }}_{0},{\hat{\alpha }}_{3},{\hat{\alpha }}_{2},{\hat{\alpha }}_{5},{\hat{\alpha }}_{4},{\hat{\alpha }}_{7},{\hat{\alpha }}_{6}\right){]}_{C_{21}C_{22}C_{23}C_{11}C_{12}C_{13}}, \end{array}$$

where

(22) $$\begin{array}{ccc} {\hat{\alpha }}_{0}=\alpha_{0}\beta_{6}, & {\hat{\alpha }}_{1}=-{\left(-1\right)}^{b_{3}}\alpha_{1}\beta_{7}, & {\hat{\alpha }}_{2}={\left(-1\right)}^{b_{2}}\alpha_{2}\beta_{4},\\ {\hat{\alpha }}_{3}=-{\left(-1\right)}^{b_{2}⊕b_{3}}\alpha_{3}\beta_{5}, & {\hat{\alpha }}_{4}=-{\left(-1\right)}^{b_{1}}\alpha_{4}\beta_{2}, & {\hat{\alpha }}_{5}={\left(-1\right)}^{b_{1}⊕b_{3}}\alpha_{5}\beta_{3},\\ {\hat{\alpha }}_{6}=-{\left(-1\right)}^{b_{1}⊕b_{2}}\alpha_{6}\beta_{0}, & {\hat{\alpha }}_{7}={\left(-1\right)}^{b_{1}⊕b_{2}⊕b_{3}}\alpha_{7}\beta_{1}. \end{array}$$

To achieve the goal, both Bob and Charlie_2_ carry out projective measurements under *X*-basis {|+〉,|−〉} and inform Charlie_1_ of the measurement outcomes in forms of classical message. Corresponding to Charlie_2_’s measurement outcome, Charlie_1_ first performs the unitary operation listed in Table 2.

To be explicit, assume Bob and Charlie_2_’s measurement outcomes are ${|+--\rangle }_{B_{1}B_{2}B_{3}}$ and ${|+-+\rangle }_{C_{21}C_{22}C_{23}}$, then the remaining qubits collapse into:

(23) $$\begin{array}{c} \frac{1}{8}(\alpha_{0}\beta_{6}|-++\rangle +\alpha_{1}\beta_{7}|-+-\rangle -\alpha_{2}\beta_{4}|--+\rangle -\alpha_{3}\beta_{5}|---\rangle \\ -\alpha_{4}\beta_{2}|+++\rangle -\alpha_{5}\beta_{3}|++-\rangle +\alpha_{6}\beta_{0}|+-+\rangle +\alpha_{7}\beta_{1}{\left|+--\rangle \right)}_{C_{11}C_{12}C_{13}}. \end{array}$$

Charlie_1_ performs the operation $H_{C_{11}}{\left(XH\right)}_{C_{12}}H_{C_{13}}$ and gets:

(24) $$\begin{array}{c} \frac{1}{8}f\left(\alpha_{6},\alpha_{7},-\alpha_{4},-\alpha_{5},-\alpha_{2},-\alpha_{3},\alpha_{0},-\alpha_{1}\right). \end{array}$$

In the following, Charlie_1_ employs similar operations to those of the assigned receiver Bob in **Case 1**. Charlie_1_ introduces three qubits with initial states ${|000\rangle }_{e_{1}e_{2}e_{3}}$ and obtains

(25) $$\begin{array}{cc} |\Gamma^{\prime }\rangle = & \frac{1}{8}{(\alpha_{0}\beta_{6}|110\rangle }^{⊗2}+\alpha_{1}\beta_{7}{|111\rangle }^{⊗2}-\alpha_{2}\beta_{4}{|100\rangle }^{⊗2}-\alpha_{3}\beta_{5}{|101\rangle }^{⊗2}\\ & -\alpha_{4}\beta_{2}{|010\rangle }^{⊗2}-\alpha_{5}\beta_{3}{|011\rangle }^{⊗2}+\alpha_{6}\beta_{0}{|000\rangle }^{⊗2}+\alpha_{7}\beta_{1}{|001\rangle }^{⊗2}{)}_{C_{11}C_{12}C_{13}e_{1}e_{2}e_{3}} \end{array}$$

after performing the CNOT operations $C_{C_{1j}e_{j}}$, j=1,2,3. Then he performs POVM defined in Equation (15). If Charlie_1_ obtains $O_{r_{1}r_{2}r_{3}}$ with equal probability $\langle \Gamma^{\prime }|O_{r_{1}r_{2}r_{3}}|\Gamma^{\prime }\rangle =\frac{1}{64\omega \varepsilon }$, he can execute appropriate Pauli operations listed in Table 3 to recover the secret state. Otherwise, he fails.

Similar discussions can be made for other measurement results of Alice, Bob and Charlie_2_. Take all the possible measurement results into account, one can find that the success probability of Charlie_1_ is identical to that in Equation (18).

## 3. Probabilistic HQIS of Arbitrary n-Qubit States with Three Agents

The above scheme for an arbitrary three-qubit state can be generalized to an arbitrary *n*-qubit state,

(26) $${|\varphi \rangle }_{1\cdots n}=\sum_{a_{1},\cdots ,a_{n}=0}^{1}\alpha_{a}{|a_{1}\cdots a_{n}\rangle }_{1\cdots n},$$

where the subscript *a* represents the decimal form of binary string $a_{1}\cdots a_{n}$, $\alpha_{a}$ is a complex number satisfying $\sum_{a=0}^{2^{n}-1}{\left|\alpha_{a}\right|}^{2}=1$.

*n* NME four-qubit cluster states $|\Psi_{j}\rangle_{A_{j}B_{j}C_{1j}C_{2j}}$ defined in Equation (2) are shared as the quantum channel, where the particle $A_{j}$ is in Alice’s possession, while the agents Bob, Charlie_1_ and Charlie_2_ possess particles $B_{j}$, $C_{1j}$, $C_{2j}$, j=1,⋯,n.

The combined state of the total system is:

(27) $$\begin{array}{cc} & {|\varphi \rangle }_{1\cdots n}⊗|\Psi_{1}\rangle_{A_{1}B_{1}C_{11}C_{21}}⊗\cdots ⊗{|\Psi_{n}\rangle }_{A_{n}B_{n}C_{1n}C_{2n}}\\ = & \sum_{a_{1},\cdots ,a_{n}=0}^{1}\alpha_{a}\prod_{j=1}^{n}(\sum_{p_{j},q_{j}=0}^{1}{\left(-1\right)}^{p_{j}q_{j}}\beta_{jp_{j}}|a_{j}p_{j}p_{j}q_{j}q_{j}\rangle {)}_{jA_{j}B_{j}C_{1j}C_{2j}}. \end{array}$$

The detailed process is described as follows.

**Step 1** Alice initially executes joint projective measurements on her qubits $(1,A_{1}),\cdots ,$ $\left(n,A_{n}\right)$ under the Bell basis. Then, she broadcasts 2n cbits $s_{1}t_{1}\cdots s_{n}t_{n}$ to announce her measurement result $|\xi_{s_{1}t_{1}}\rangle_{1A_{1}}\cdots {|\xi_{s_{n}t_{n}}\rangle }_{nA_{n}}$.

Since

(28) $$|kh\rangle =\frac{1}{\sqrt{2}}\sum_{n=0}^{1}{\left(-1\right)}^{kn}|\xi_{n,k⊕h}\rangle ,$$

the state collapses into

(29) $$|g_{s_{1}t_{1}\cdots s_{n}t_{n}}\rangle =\frac{1}{2^{n/2}}\sum_{a_{1},\cdots ,a_{n}=0}^{1}\alpha_{a}\prod_{j=1}^{n}(\sum_{q_{j}=0}^{1}{\left(-1\right)}^{a_{j}\left(s_{j}⊕q_{j}\right)⊕t_{j}q_{j}}\beta_{j,a_{j}⊕t_{j}}|a_{j}⊕t_{j}\rangle |q_{j}\rangle |q_{j}{\rangle )}_{B_{j}C_{1j}C_{2j}}.$$

**Step 2** According to Alice’s measurement result, the agents perform the appropriate operations to realize that any one of agents recovers the secret state.

Due to the different grades of agents, we still discuss it in two cases.

**Case 1** The high-grade agent is designated as the receiver.

The high-grade agent Bob only requires the help of one low-grade agent as Charlie_1_ and Charlie_2_ have exactly the same qubits. If one of the low-grade agents performs *n* single-qubit projective measurements under the *Z*-basis and obtains the measurement result $|c_{1}\rangle \cdots |c_{n}\rangle$ which is sent to Bob in the form of classical bits $c_{1}\cdots c_{n}$, Bob’s qubits collapse into

(30) $$\frac{1}{2^{n/2}}\sum_{a_{1},\cdots ,a_{n}=0}^{1}\alpha_{a}\prod_{j=1}^{n}{\left(-1\right)}^{a_{j}\left(s_{j}⊕c_{j}\right)}\beta_{j,a_{j}⊕t_{j}}|a_{j}⊕t_{j}\rangle .$$

Bob introduces *n* auxiliary qubits ${|0\cdots 0\rangle }_{e_{1}\cdots e_{n}}$ and performs CNOT operations $C_{B_{j}e_{j}}$. Thus, he gets

(31) $$|\Gamma \rangle =\frac{1}{2^{n/2}}\sum_{a_{1},\cdots ,a_{n}=0}^{1}\alpha_{a}\prod_{j=1}^{n}{\left(-1\right)}^{a_{j}\left(s_{j}⊕c_{j}\right)}\beta_{j,a_{j}⊕t_{j}}{|a_{j}⊕t_{j}\rangle }_{B_{j}}{|a_{j}⊕t_{j}\rangle }_{e_{j}}.$$

The state in the above equation can be decomposed into:

(32) $$|\Gamma \rangle =\frac{1}{2^{3n/2}}\sum_{r_{1},\cdots ,r_{n}=0}^{1}{|K_{r_{1}\cdots r_{n}}\rangle }_{B_{1}\cdots B_{n}}{|Q_{r_{1}\cdots r_{n}}\rangle }_{e_{1}\cdots e_{n}},$$

where

(33) $$|K_{r_{1}\cdots r_{n}}\rangle =\sum_{a_{1},\cdots ,a_{n}=0}^{1}\alpha_{a}\prod_{j=1}^{n}{\left(-1\right)}^{a_{j}\left(s_{j}⊕c_{j}⊕r_{j}\right)⊕t_{j}r_{j}}{|a_{j}⊕t_{j}\rangle }_{B_{j}}$$

and

(34) $$|Q_{r_{1}\cdots r_{n}}\rangle =\sum_{a_{1},\cdots ,a_{n}=0}^{1}\prod_{j=1}^{n}{\left(-1\right)}^{a_{j}r_{j}}\beta_{ja_{j}}{|a_{j}\rangle }_{e_{j}}.$$

Since $|Q_{r_{1}\cdots r_{n}}\rangle ,r_{1},\cdots ,r_{n}=0,1$ are not mutually orthogonal in general, Bob had better perform POVM which plays an important role in state discrimination. The measurement operators are:

(35) $$O_{r_{1}\cdots r_{n}}=\frac{1}{\omega }|M_{r_{1}\cdots r_{n}}\rangle \langle M_{r_{1}\cdots r_{n}}|,\;O_{2^{n}}=I-\sum_{r_{1}\cdots r_{n}=0}^{1}O_{r_{1}\cdots r_{n}},$$

where ω is related to the coefficient of the quantum channel and

(36) $$|M_{r_{1}\cdots r_{n}}\rangle =\frac{1}{\sqrt{\varepsilon }}\sum_{a_{1},\cdots ,a_{n}=0}^{1}\prod_{j=1}^{n}{\left(-1\right)}^{a_{j}r_{j}}\frac{1}{\beta_{ja_{j}}}|a_{j}\rangle .$$

Here, $\varepsilon =\sum_{a_{1},\cdots ,a_{n}=0}^{1}\frac{1}{\beta_{1a_{1}}^{2}\cdots \beta_{na_{n}}^{2}}$ and ω satisfies $\omega \geq 4^{n}\max\left\{\beta_{0}^{2},\cdots ,\beta_{2^{n}-1}^{2}\right\}$ which is to meet the condition that the elements of POVM must be positive operators. If Bob obtains measurement result $O_{r_{1}\cdots r_{n}}$ with equal probability $\langle \Gamma |O_{r_{1}\cdots r_{n}}|\Gamma \rangle =\frac{1}{2^{n}\omega \varepsilon }$, his qubits $\left({B_{1},\cdots ,B}_{n}\right)$ collapse to $|K_{r_{1}\cdots r_{n}}\rangle$. Then, Bob performs

(37) $$R_{B}={\left(Z^{s_{1}⊕c_{1}⊕r_{1}}X^{t_{1}}\right)}_{B_{1}}\cdots {\left(Z^{s_{n}⊕c_{n}⊕r_{n}}X^{t_{n}}\right)}_{B_{n}}$$

and recovers the target state. If his measurement outcome is $O_{2^{n}}$, he fails.

**Case 2** The low-grade agent is designated as the receiver.

Suppose Charlie_1_ is assigned as the receiver. Bob and Charlie_2_ need to perform projective measurements under the *X*-basis and inform him of the measurement outcomes. Since

(38) $$|h\rangle =\sum_{l=0}^{1}{\left(-1\right)}^{lh}|\tilde{l}\rangle ,\;h=0,1,$$

Charlie_1_’s qubits collapse into:

(39) $${\left(-1\right)}^{\sum_{j=1}^{n}t_{j}b_{j}}\frac{1}{2^{n}}\sum_{a_{1},\cdots ,a_{n}=0}^{1}\alpha_{a}\prod_{j=1}^{n}{\left(-1\right)}^{a_{j}\left(s_{j}⊕b_{j}\right)}\beta_{j,a_{j}⊕t_{j}}{|\tilde{a_{j}⊕t_{j}⊕c_{j}}\rangle }_{C_{1j}}.$$

If Bob’s and Charlie_2_’s measurement outcomes are $|\tilde{b_{j}}\rangle$ and $|\tilde{c_{j}}\rangle$, $b_{j},c_{j}\in \left\{0,1\right\}$, j=1,⋯,n. Charlie_1_ performs ${\left(X^{c_{1}}H\right)}_{C_{11}}\cdots {\left(X^{c_{n}}H\right)}_{C_{1n}}$ and gets:

(40) $${\left(-1\right)}^{\sum_{j=1}^{n}t_{j}b_{j}}\frac{1}{2^{n}}\sum_{a_{1},\cdots ,a_{n}=0}^{1}\alpha_{a}\prod_{j=1}^{n}{\left(-1\right)}^{a_{j}\left(s_{j}⊕b_{j}\right)}\beta_{j,a_{j}⊕t_{j}}{|a_{j}⊕t_{j}\rangle }_{C_{1j}}.$$

Similar to **Case 1**, Charlie_1_ carries out POVM in Equation (35) after introducing auxiliary qubits and performing CNOT operations. If his measurement outcome is $O_{r_{1}\cdots r_{n}}$, Charlie_1_ can reconstruct the secret state by performing recovery operation

(41) $$R_{C}={\left(Z^{s_{1}⊕b_{1}⊕r_{1}}X^{t_{1}}\right)}_{C_{11}}\cdots {\left(Z^{s_{n}⊕b_{n}⊕r_{n}}X^{t_{n}}\right)}_{C_{1n}}.$$

While measurement result $O_{2^{n}}$ indicates the task is failed.

Regardless of the grade of agents, the total success probability is:

(42) $$P=\frac{2^{n}\times 4^{n}}{\omega \varepsilon }=\frac{2^{3n}{\left(\beta_{10}\beta_{11}\cdots \beta_{n0}\beta_{n1}\right)}^{2}}{\omega }.$$

Since $|\beta_{j0}\left|\leq \right|\beta_{j1}|$, the total success probability is

(43) $$P\leq 4^{n}\beta_{10}^{2}\cdots \beta_{n0}^{2}.$$

## 4. Probabilistic HQIS of Arbitrary n-Qubit States with N Agents

It is necessary to investigate the situation of multiple agents as there are many users in a practical communication network. The hierarchy of agents may be diverse. Here, we consider one simple case in which the *N* agents are divided into two grades: *u* agents (Bob_1_, ⋯, Bob*_u_*) lie in high grade and *v* agents (Charlie_1_, ⋯, Charlie*_v_*) lie in low grade.

The main aim is teleporting an arbitrary *n*-qubit state ${|\varphi \rangle }_{1\cdots n}$ shown in Equation (26) from the sender to any one of the *N* agents in an asymmetrical way.

**Figure 1:**
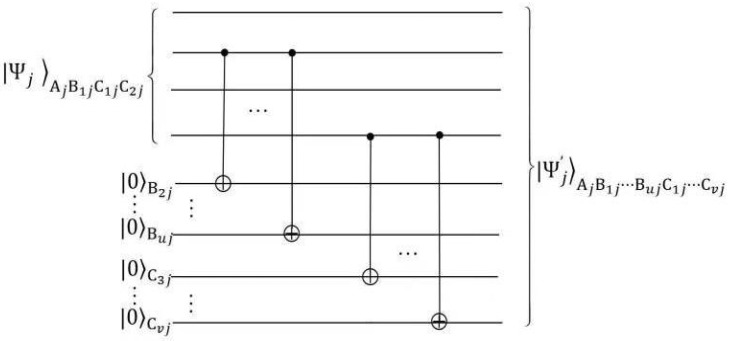
The generation circuit of the state $|\Psi_{j}^{\prime }\rangle$.

Before the scheme officially begins, Alice prepares *n* NME four-qubit cluster states $|\Psi_{j}\rangle_{A_{j}B_{1j}C_{1j}C_{2j}}$, j=1,⋯,n and introduces some qubits ${|0\cdots 0\rangle }_{B_{2j}\cdots B_{uj}C_{3j}\cdots C_{vj}}$. Then she carries out a series of CNOT operations as shown in Figure 1 and gets

(44) $$|\Psi_{j}^{\prime }\rangle =(\beta_{j0}|0\rangle |\psi^{0}\rangle +\beta_{j1}\left|1\rangle \right|\psi^{1}{\rangle )}_{A_{j}B_{1j}\cdots B_{uj}C_{1j}\cdots C_{vj}},\;j=1,\cdots ,n,$$

where

(45) $$|\psi^{0}\rangle =|\underset{u}{\underset{︸}{0\cdots 0}}\underset{v}{\underset{︸}{0\cdots 0}}\rangle +|\underset{u}{\underset{︸}{0\cdots 0}}\underset{v}{\underset{︸}{1\cdots 1}}\rangle ,\;|\psi^{1}\rangle =|\underset{u}{\underset{︸}{1\cdots 1}}\underset{v}{\underset{︸}{0\cdots 0}}\rangle -|\underset{u}{\underset{︸}{1\cdots 1}}\underset{v}{\underset{︸}{1\cdots 1}}\rangle .\;$$

**Figure 2:**
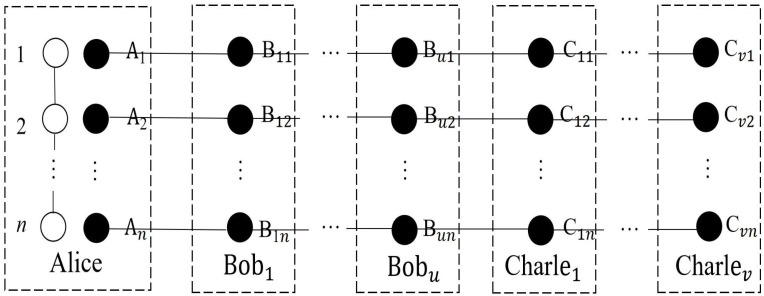
The qubit shared by the sender and *N* agents. White points represent Alice’s secret particles, black points represent particles in the NME cluster states. The solid lines stand for entanglements.

Next, Alice distributes the qubits $B_{1j},\cdots ,B_{uj}$, $C_{1j},\cdots ,C_{vj}$ to Bob_1_, ⋯, Bob*_u_*, Charlie_1_, ⋯, and Charlie*_v_*, respectively. The distribution of qubits among *N* agents is shown in Figure 2.

The initial whole system is:

(46) $$\begin{array}{cc} & {|\varphi \rangle }_{12\cdots n}⊗|\Psi_{1}^{\prime }\rangle_{A_{1}B_{11}\cdots B_{u1}C_{11}\cdots C_{v1}}⊗\cdots ⊗{|\Psi_{n}^{\prime }\rangle }_{A_{n}B_{1n}\cdots B_{un}C_{1n}\cdots C_{vn}}\\ = & \sum_{a_{1},\cdots ,a_{n}=0}^{1}\alpha_{a}\prod_{j=1}^{n}(\sum_{p_{j},q_{j}=0}^{1}{\left(-1\right)}^{p_{j}q_{j}}\beta_{jp_{j}}|a_{j}p_{j}\cdots p_{j}q_{j}\cdots q_{j}\rangle {)}_{jA_{j}B_{1j}\cdots B_{uj}C_{1j}\cdots C_{vj}}. \end{array}$$

The scheme can be illustrated as follows.

**Step 1** The sender Alice carries out Bell-basis measurements on her qubits $(1,A_{1}),\cdots ,$$\left(n,A_{n}\right)$. If the measurement result is $|\xi_{s_{1}t_{1}}\rangle ,\cdots ,|\xi_{s_{n}t_{n}}\rangle$, she broadcasts classical bits $s_{1}t_{1}\cdots s_{n}t_{n}$, $s_{j},t_{j}\in \left\{0,1\right\}$, j=1,⋯,n.

Since the state in Equation (46) can be rewritten as:

(47) $$\begin{array}{c} \frac{1}{2^{n/2}}\sum_{a_{1},\cdots ,a_{n}=0}^{1}\alpha_{a}\prod_{j=1}^{n}(\sum_{q_{j},s_{j},t_{j}=0}^{1}{\left(-1\right)}^{a_{j}\left(s_{j}⊕q_{j}\right)⊕t_{j}q_{j}}\beta_{j,a_{j}⊕t_{j}}|\xi_{s_{j}t_{j}}\rangle |a_{j}⊕t_{j}\rangle^{⊗u}|q_{j}\rangle^{⊗v}), \end{array}$$

the state collapses to:

(48) $$\begin{array}{cc} |g_{s_{1}t_{1}\cdots s_{n}t_{n}}\rangle = & \frac{1}{2^{n/2}}\sum_{a_{1},\cdots ,a_{n}=0}^{1}\alpha_{a}\prod_{j=1}^{n}(\sum_{q_{j}=0}^{1}{\left(-1\right)}^{a_{j}\left(s_{j}⊕q_{j}\right)⊕t_{j}q_{j}}\beta_{j,a_{j}⊕t_{j}}|a_{j}⊕t_{j}\rangle^{⊗u}|q_{j}\rangle^{⊗v}). \end{array}$$

**Step 2** According to Alice’s classical message, the agents apply the appropriate operations to achieve the goal that any one of agents recovers the secret state.

Since the same-grade agents have the same authority, we assume that the high-grade agent Bob_1_ or the low-grade agent Charlie_1_ recovers the secret state.

**Case 1** The high-grade agent Bob_1_ is designated as the receiver.

The state in Equation (48) can be rewritten as:

(49) $$\begin{array}{c} \sum_{a_{1},\cdots ,a_{n}=0}^{1}\alpha_{a}\prod_{j=1}^{n}\{\sum_{q_{j}=0}^{1}{\left(-1\right)}^{a_{j}\left(s_{j}⊕q_{j}\right)⊕t_{j}q_{j}}|a_{j}⊕t_{j}\rangle \prod_{t=2}^{u}(\sum_{k_{lj}=0}^{1}{\left(-1\right)}^{\left(a_{j}⊕t_{j}\right)l_{tj}}\beta_{j,a_{j}⊕t_{j}}|\tilde{k_{lj}}\rangle )|q_{j}\rangle^{⊗v}\} \end{array}$$

up to the global phase.

Only if the other high-grade agent and one low-grade agent respectively perform projective measurements under the *X*-basis and *Z*-basis, can Bob_1_ restore the target state with a certain probability. Assume the measurement results of Bob_2_, ⋯, Bob*_u_* are $|\tilde{b_{2j}}\rangle ,\cdots ,|\tilde{b_{uj}}\rangle$ and the measurement outcomes of Charlie_1_ are $|c_{j}\rangle$, where $b_{2j},\cdots ,b_{uj}$, $c_{j}\in \left\{0,1\right\}$, j=1,⋯,n. At the moment, Bob_1_’s qubits collapse into:

(50) $$\begin{array}{c} \sum_{a_{1},\cdots ,a_{n}=0}^{1}\alpha_{a}\prod_{j=1}^{n}{\left(-1\right)}^{a_{j}\left(s_{j}⊕c_{j}⊕b_{2j}\cdots ⊕b_{uj}\right)}\beta_{j,a_{j}⊕t_{j}}|a_{j}⊕t_{j}\rangle \end{array}$$

up to the global phase.

Bob_1_ introduces auxiliary qubits ${|0\cdots 0\rangle }_{e_{1}\cdots e_{n}}$ and performs CNOT operations $C_{B_{j}e_{j}}$. Thus, he gets:

(51) $$\sum_{a_{1},\cdots ,a_{n}=0}^{1}\alpha_{a}\prod_{j=1}^{n}{\left(-1\right)}^{a_{j}\left(s_{j}⊕c_{j}⊕b_{2j}\cdots ⊕b_{uj}\right)}\beta_{j,a_{j}⊕t_{j}}|a_{j}⊕t_{j}\rangle_{B_{j}}{|a_{j}⊕t_{j}\rangle }_{e_{j}}.$$

Then he performs POVM in Equation (35) on the auxiliary qubits. Analogously, if his POVM outcome is $O_{r_{1}\cdots r_{n}}$, Bob_1_ carries out unitary operation

(52) $$R_{B}={\left(Z^{c_{1}⊕s_{1}⊕b_{21}⊕\cdots ⊕b_{u1}⊕r_{1}}X^{t_{1}}\right)}_{B_{11}}\cdots {\left(Z^{c_{n}⊕s_{n}⊕b_{2n}⊕\cdots ⊕b_{un}⊕r_{n}}X^{t_{n}}\right)}_{B_{1n}}$$

and restores the secret state on his own qubits. If he obtains measurement result $O_{2^{n}}$, the goal cannot be achieved.

**Case 2** The low-grade agent Charlie_1_ is designated as the receiver.

To assist Charlie_1_ in recovering the target state, all the other agents perform projective measurements under the *X*-basis and inform him of the measurement outcomes. Assume the measurement outcomes of Bob_1_, ⋯, Bob*_u_*, Charlie_2_, ⋯ Charlie*_v_* are $|\tilde{b_{1j}}\rangle ,\cdots ,|\tilde{b_{uj}}\rangle$, $|\tilde{c_{2j}}\rangle ,\cdots ,|\tilde{c_{vj}}\rangle$, j=1,⋯,n. Then Charlie_1_’s qubits collapse into:

(53) $$\begin{array}{c} \sum_{a_{1},\cdots ,a_{n}=0}^{1}\alpha_{a}\prod_{j=1}^{n}{\left(-1\right)}^{a_{j}\left(s_{j}⊕b_{1j}⊕\cdots ⊕b_{uj}\right)}\beta_{j,a_{j}⊕t_{j}}{|\tilde{a_{j}⊕t_{j}⊕c_{2j}⊕\cdots ⊕c_{vj}}\rangle }_{C_{1j}} \end{array}$$

up to a global phase.

Charlie_1_ performs ${\left(X^{c_{21}⊕\cdots ⊕c_{v1}}H\right)}_{C_{11}}\cdots {\left(X^{c_{2n}⊕\cdots ⊕c_{vn}}H\right)}_{C_{1n}}$ and gets

(54) $$\sum_{a_{1},\cdots ,a_{n}=0}^{1}\alpha_{a}\prod_{j=1}^{n}{\left(-1\right)}^{a_{j}\left(s_{j}⊕b_{1j}⊕\cdots ⊕b_{uj}\right)}\beta_{j,a_{j}⊕t_{j}}{|a_{j}⊕t_{j}\rangle }_{C_{1j}}.$$

Similar to **Case 1**, Charlie_1_ performs POVM in Equation (35) after introducing auxiliary qubits and executing CNOT operations. If the measurement outcome is $O_{r_{1}\cdots r_{n}}$, his recovery operation can be summarized as:

(55) $$R_{C}={\left(Z^{s_{1}⊕b_{11}⊕\cdots ⊕b_{u1}⊕r_{1}}X^{t_{1}⊕c_{21}⊕\cdots ⊕c_{v1}}H\right)}_{C_{11}}\cdots {\left(Z^{s_{n}⊕b_{1n}⊕\cdots ⊕b_{un}⊕r_{n}}X^{t_{1}⊕c_{2n}⊕\cdots ⊕c_{vn}}H\right)}_{C_{1n}}.$$

While measurement result $O_{2^{n}}$ indicates task is failed.

The total success probability is the same as the case of three agents.

## 5. Security Analysis

For simplicity, take the case of three agents as an example. In order to ensure the security, Alice adopts the following strategies to detect eavesdropping. Besides *n* NME four-qubit cluster states $|\Psi_{j}\rangle_{A_{j}B_{j}C_{1j}C_{2j}}$, j=1,⋯,n, Alice generates an ordered sequence $P_{A}=\left\{A_{1},\cdots ,A_{n}\right\}$ using all the first qubits in her possession. Similarly, she forms another three ordered sequences $P_{B}=\left\{B_{1},\cdots ,B_{n}\right\}$, $P_{C_{1}}=\left\{C_{11},\cdots ,C_{1n}\right\}$ and $P_{C_{2}}=\left\{C_{21},\cdots ,C_{2n}\right\}$. Then she prepares 3n decoy photons $\left\{d_{1},\cdots ,d_{3n}\right\}$ chosen from {|0〉,|1〉,|+〉,|−〉} and randomly insert them into the three sequence to yield the larger sequences $P_{B}^{\prime }$, $P_{C_{1}}^{\prime }$, $P_{C_{2}}^{\prime }$. It should be emphasized that the final order of all sequences is only known by Alice.

There are two kinds of eavesdropping. One is that an illegal external eavesdropper Eve manages to steal secret information, the other is that there is a dishonest internal eavesdropper.

**(I)** Outsider attack.

It is not determined which agent is the receiver until the entangled channel particles are distributed. In order to obtain the secret state, Eve intercepts all particles distributed from Alice to the three agents and resends the forged one to them. In this case, once Alice conducts eavesdropping inspection, she tells the three agents about the location, state and measurement basis (*Z*-basis or *X*-basis). Then she asks each agent to measure the decoy photons under the measurement basis and publish the measurement results. Only if the agents’ measurement results are consistent with Alice’s predicted results, does the protocol continue. In addition, since Eve has no access to the coefficients of the NME channel, he can not carry out correct POVM to restore the secret state even if he intercepts the agents’ particles.

**(II)** Insider attack.

The influence of dishonest agents is destructive. Without loss of generality, assume that high-grade agent Bob is the internal eavesdropper. If everyone completely ignores his identity and consents to him as the receiver, he can easily acquire the secret state. In this case, our agreement cannot resist internal attacks. If the low-grade agent is designated as the receiver and Bob wants to steal the confidential state, one way is to intercept the channel particles of the low-grade agents and resend them false particles. However, because the decoy photons of different agents are different, eavesdropping will be found by the eavesdropping inspection. Another way is an entanglement measurement attack. Bob performs the unitary operation to act on Charlie_1_’s or Charlie_2_’s particles he has intercepted and the auxiliary particles he has prepared, so that he can measure the auxiliary particles to obtain information about the secret state. This attack will also be detected because of errors caused by decoy particles. The specific process is similar to that in Ref. [37].

## 6. Discussions and Conclusions

In fact, besides POVM, the designated receiver can adopt the collective unitary operation to restore the secret state. Taking the high-grade agent Bob as the receiver, based on one of the low-grade agent’s help, Bob’s qubits collapse into the state in Equation (30). Similar to Ref. [34], Bob first introduces an auxiliary qubit ${|0\rangle }_{e}$ and performs a collective unitary transformation $\mathrm{diag}(U_{0},\cdots ,U_{2^{n}-1})$ on his qubits $\left(B_{1},\cdots ,B_{n},e\right)$. Then Bob measures the auxiliary qubit under the *Z*-basis. If the measurement result is ${|0\rangle }_{e}$, he performs appropriate operations on the collapsed state and recovers the target state. If the measurement result is ${|1\rangle }_{e}$, the protocol fails.

In conclusion, we devise a universal scheme to probabilistically realize the HQIS of arbitrary multi-qubit states among multiple agents by using NME four-qubit cluster states as the quantum resource. Comparing with the HQIS scheme for arbitrary single- and two-qubit states [22,23], our work has the following advantages: (1) Our schemes are applicable for arbitrary multi-qubit states with the aid of elaborately constructed multi-qubit POVM; (2) We derive the general expression of a recovery operation which clearly discloses the relationship with the measurement results; (3) We describe the specific process of HQIS with multiple agents. Our schemes are practical as NME states are easier to generate and maintain than the maximally entangled resources and agents’ unequal authorities are in line with the actual communication. Moreover, the preparation of the NME four-qubit cluster states, Bell-basis measurement, local CNOT and Pauli operations and POVM are required in our schemes. Besides POVM, other operations are easily implemented. Fortunately, POVM can be realized by using measurement assisted programmable quantum processors [38] and the ensemble approach to polarization optics [39]. It means our schemes are achievable since these necessary operations are feasible under the current experimental technologies.

## Figures and Tables

**Table 1:** Bob’s recovery operations (BRO) depending on one low-grade agent’s measurement outcome (LAMO) and his own POVM outcome $O_{r_{1}r_{2}r_{3}}$.

LAMO	$r_{1}r_{2}r_{3}$	BRO	$r_{1}r_{2}r_{3}$	BRO
|000〉	000	$Y_{B_{1}}X_{B_{2}}Z_{B_{3}}$	100	$X_{B_{1}}X_{B_{2}}Z_{B_{3}}$
	001	$Y_{B_{1}}X_{B_{2}}I_{B_{3}}$	101	$X_{B_{1}}X_{B_{2}}I_{B_{3}}$
	010	$Y_{B_{1}}Y_{B_{2}}Z_{B_{3}}$	110	$X_{B_{1}}Y_{B_{2}}Z_{B_{3}}$
	011	$Y_{B_{1}}Y_{B_{2}}I_{B_{3}}$	111	$X_{B_{1}}Y_{B_{2}}I_{B_{3}}$
|001〉	000	$Y_{B_{1}}X_{B_{2}}I_{B_{3}}$	100	$X_{B_{1}}X_{B_{2}}I_{B_{3}}$
	001	$Y_{B_{1}}X_{B_{2}}Z_{B_{3}}$	101	$X_{B_{1}}X_{B_{2}}Z_{B_{3}}$
	010	$Y_{B_{1}}Y_{B_{2}}I_{B_{3}}$	110	$X_{B_{1}}Y_{B_{2}}I_{B_{3}}$
	011	$Y_{B_{1}}Y_{B_{2}}Z_{B_{3}}$	111	$X_{B_{1}}Y_{B_{2}}Z_{B_{3}}$
|010〉	000	$Y_{B_{1}}Y_{B_{2}}Z_{B_{3}}$	100	$X_{B_{1}}Y_{B_{2}}Z_{B_{3}}$
	001	$Y_{B_{1}}Y_{B_{2}}I_{B_{3}}$	101	$X_{B_{1}}Y_{B_{2}}I_{B_{3}}$
	010	$Y_{B_{1}}X_{B_{2}}Z_{B_{3}}$	110	$X_{B_{1}}X_{B_{2}}Z_{B_{3}}$
	011	$Y_{B_{1}}X_{B_{2}}I_{B_{3}}$	111	$X_{B_{1}}X_{B_{2}}I_{B_{3}}$
|011〉	000	$Y_{B_{1}}Y_{B_{2}}I_{B_{3}}$	100	$X_{B_{1}}Y_{B_{2}}I_{B_{3}}$
	001	$Y_{B_{1}}Y_{B_{2}}Z_{B_{3}}$	101	$X_{B_{1}}Y_{B_{2}}Z_{B_{3}}$
	010	$Y_{B_{1}}X_{B_{2}}I_{B_{3}}$	110	$X_{B_{1}}X_{B_{2}}I_{B_{3}}$
	011	$Y_{B_{1}}X_{B_{2}}Z_{B_{3}}$	111	$X_{B_{1}}X_{B_{2}}Z_{B_{3}}$
|100〉	000	$X_{B_{1}}X_{B_{2}}Z_{B_{3}}$	100	$Y_{B_{1}}X_{B_{2}}Z_{B_{3}}$
	001	$X_{B_{1}}X_{B_{2}}I_{B_{3}}$	101	$Y_{B_{1}}X_{B_{2}}I_{B_{3}}$
	010	$X_{B_{1}}Y_{B_{2}}Z_{B_{3}}$	110	$Y_{B_{1}}Y_{B_{2}}Z_{B_{3}}$
	011	$X_{B_{1}}Y_{B_{2}}I_{B_{3}}$	111	$Y_{B_{1}}Y_{B_{2}}I_{B_{3}}$
|101〉	000	$X_{B_{1}}X_{B_{2}}I_{B_{3}}$	100	$Y_{B_{1}}X_{B_{2}}I_{B_{3}}$
	001	$X_{B_{1}}X_{B_{2}}Z_{B_{3}}$	101	$Y_{B_{1}}X_{B_{2}}Z_{B_{3}}$
	010	$X_{B_{1}}Y_{B_{2}}I_{B_{3}}$	110	$Y_{B_{1}}Y_{B_{2}}I_{B_{3}}$
	011	$X_{B_{1}}Y_{B_{2}}Z_{B_{3}}$	111	$Y_{B_{1}}Y_{B_{2}}Z_{B_{3}}$
|110〉	000	$X_{B_{1}}Y_{B_{2}}Z_{B_{3}}$	100	$Y_{B_{1}}Y_{B_{2}}Z_{B_{3}}$
	001	$X_{B_{1}}Y_{B_{2}}I_{B_{3}}$	101	$Y_{B_{1}}Y_{B_{2}}I_{B_{3}}$
	010	$X_{B_{1}}X_{B_{2}}Z_{B_{3}}$	110	$Y_{B_{1}}X_{B_{2}}Z_{B_{3}}$
	011	$X_{B_{1}}X_{B_{2}}I_{B_{3}}$	111	$Y_{B_{1}}X_{B_{2}}I_{B_{3}}$
|111〉	000	$X_{B_{1}}Y_{B_{2}}I_{B_{3}}$	100	$Y_{B_{1}}Y_{B_{2}}I_{B_{3}}$
	001	$X_{B_{1}}Y_{B_{2}}Z_{B_{3}}$	101	$Y_{B_{1}}Y_{B_{2}}Z_{B_{3}}$
	010	$X_{B_{1}}X_{B_{2}}I_{B_{3}}$	110	$Y_{B_{1}}X_{B_{2}}I_{B_{3}}$
	011	$X_{B_{1}}X_{B_{2}}Z_{B_{3}}$	111	$Y_{B_{1}}X_{B_{2}}Z_{B_{3}}$

**Table 2:** Charlie_1_’s operations depending on Charlie_2_’s measurement outcome (MO).

Charlie_2_’s MO	Charlie_1_’s Operation
|+++〉	$H_{C_{11}}H_{C_{12}}H_{C_{13}}$
|++−〉	$H_{C_{11}}H_{C_{12}}{\left(XH\right)}_{C_{13}}$
|+−+〉	$H_{C_{11}}{\left(XH\right)}_{C_{12}}H_{C_{13}}$
|+−−〉	$H_{C_{11}}{\left(XH\right)}_{C_{12}}{\left(XH\right)}_{C_{13}}$
|−++〉	${\left(XH\right)}_{C_{11}}H_{C_{12}}H_{C_{13}}$
|−+−〉	${\left(XH\right)}_{C_{11}}H_{C_{12}}{\left(XH\right)}_{C_{13}}$
|−−+〉	${\left(XH\right)}_{C_{11}}{\left(XH\right)}_{C_{12}}H_{C_{13}}$
|−−−〉	${\left(XH\right)}_{C_{11}}{\left(XH\right)}_{C_{12}}{\left(XH\right)}_{C_{13}}$

**Table 3:** Charlie_1_’s recovery operation (CRO) depending on his own POVM measurement outcome $O_{r_{1}r_{2}r_{3}}$ when Bob and Charlie_2_’s outcomes are ${|+--\rangle }_{B_{1}B_{2}B_{3}}$ and ${|+-+\rangle }_{C_{21}C_{22}C_{23}}$.

$r_{1}r_{2}r_{3}$	CRO	$r_{1}r_{2}r_{3}$	CRO
000	$Y_{C_{11}}Y_{C_{12}}I_{C_{13}}$	100	$X_{C_{11}}Y_{C_{12}}I_{C_{13}}$
001	$Y_{C_{11}}Y_{C_{12}}Z_{C_{13}}$	101	$X_{C_{11}}Y_{C_{12}}Z_{C_{13}}$
010	$Y_{C_{11}}X_{C_{12}}I_{C_{13}}$	110	$X_{C_{11}}X_{C_{12}}I_{C_{13}}$
011	$Y_{C_{11}}X_{C_{12}}Z_{C_{13}}$	111	$X_{C_{11}}X_{C_{12}}Z_{C_{13}}$

## Data Availability

Not applicable.
